# KELIM PSA as a Prognostic Biomarker in Castration-Resistant Prostate Cancer Treated with ARPI

**DOI:** 10.3390/jcm14228114

**Published:** 2025-11-16

**Authors:** Fatih Atalah, Fatih Kuş, Aydın Acarbay, Akgün Karakök, Onur Alkan, İsmail Nazlı, Utku Özilice, Mehmet Beşiroğlu, Mahmut Gümüş

**Affiliations:** 1 Department of Medical Oncology, Faculty of Medicine, Istanbul Medeniyet University, 34956 Istanbul, Turkey; 2 Ankara Atatürk Sanatorium Training and Research Hospital, 06290 Ankara, Turkey; 3 Department of Internal Medicine, Faculty of Medicine, Alanya Alaaddin Keykubat University, 07425 Alanya, Turkey

**Keywords:** ARPI, castration-resistant prostate cancer, KELIM PSA, prognosis, prostate cancer, PSA kinetics

## Abstract

**Background/Objectives:** Prostate cancer is a leading cause of cancer-related morbidity and mortality. While prostate-specific antigen (PSA) is crucial for monitoring, its static levels are limited in predicting outcomes precisely. The Kinetics of Elimination of PSA (KELIM PSA) has recently emerged as a dynamic biomarker of treatment response. This research sought to determine the predictive power of KELIM PSA in castration-resistant prostate cancer (CRPC) on androgen receptor pathway inhibitors (ARPI). **Methods:** This study retrospectively analyzed 98 CRPC patients treated with enzalutamide or abiraterone. The patients were categorized as either unfavorable (KELIM < 1) or favorable (KELIM ≥ 1). Demographic and clinical characteristics were compared, and survival outcomes were evaluated using Kaplan–Meier curves and Cox regression. **Results:** Of the cohort, 42 (42.9%) patients had favorable and 56 (57.1%) unfavorable KELIM values. The unfavorable group had a higher mortality rate (62.5% vs. 38.1%, *p* = 0.029). Univariate analysis showed that poor KELIM results increased mortality risk twofold (hazard ratio [HR]: 2.30, 95% confidence interval [CI]: 1.26–4.19, *p* = 0.006). In multivariable analysis, unfavorable KELIM remained independently associated with worse overall survival (HR: 2.09, 95% CI: 1.12–3.89, *p* = 0.020), together with second-line ARPI (HR: 3.19, 95% CI: 1.71–5.93, *p* < 0.001) and ADT + docetaxel during CSPC (HR: 2.14, 95% CI: 1.11–4.12, *p* = 0.022). Kaplan–Meier curves revealed that the unfavorable group had notably reduced overall survival and progression-free survival (log-rank *p* = 0.018). **Conclusions:** KELIM PSA is an independent predictor in ARPI-treated CRPC. By integrating PSA kinetics into prognostic models, risk stratification may be improved, and this may guide individualized treatment. Prospective multicenter validation is warranted.

## 1. Introduction

Prostate cancer is still a major cause of cancer-related morbidity and mortality in men worldwide, representing a serious public health issue due to its prevalence and the complexity of management [1]. Treatment strategies in prostate cancer also interact with biomarker dynamics. Survival rates have improved by adding docetaxel or abiraterone to androgen deprivation therapy (ADT) in metastatic castration-resistant prostate cancer (mCRPC) [2]. Nevertheless, outcomes remain heterogeneous, and the need for second-line treatments in CRPC indicates a notably increased risk of mortality, underlining the complex nature of the disease and treatment resistance. Furthermore, sites of metastatic involvement contribute significantly to prognosis. Favorable outcomes include regional lymph node metastasis, whereas non-regional nodal and visceral metastases suggest aggressive disease and worse survival [3].

Prostate-specific antigen (PSA) has been a fundamental tool in risk stratification for decades, and nowadays aids in diagnosis, monitoring, and prognostication. Tumor aggressiveness, volume, and histologic grading relate to PSA levels, underscoring their utility not only in diagnosis but also in predicting disease progression [4]. However, the clinical value of PSA is limited when interpreted in isolation, as factors such as age, body mass index (BMI), and treatment history can significantly influence PSA dynamics [5].

To address these limitations, attention has increasingly shifted toward PSA kinetics rather than static values. Kinetic modeling provides insights into how PSA levels change over time, along with their connection with treatment effectiveness and life expectancy. The elimination constant, the kinetics of elimination of PSA (KELIM PSA), evaluates the PSA decline rate after treatment initiation. Patients with unfavorable KELIM PSA (<1) often have worse survival than those with favorable levels, indicating tumor resistance and treatment failure [6].

Recent research affirms the prognostic value of PSA kinetics across various treatments. Wang et al. [7] found that the PSA kinetics had prognostic importance in patients with metastatic hormone-sensitive prostate cancer treated with novel hormonal therapy agents. These results align with the biological rationale that dynamic PSA parameters indicate tumor response at different stages. Within the metastatic castration-resistant prostate cancer (mCRPC) context, the FIRSTANA study [8] demonstrated KELIM PSA’s reliability as a prognostic biomarker. Unfavorable KELIM was found to be associated with considerably shorter progression-free survival (PFS) and overall survival (OS), regardless of previous taxane exposure in the castration-sensitive setting [8]. Likewise, in earlier hormone-sensitive settings, modeling PSA elimination (KELIM) and production constants (KPROD) proved predictive for PFS and OS in patients given ADT ± docetaxel [6]. These findings emphasize that kinetic parameters might improve standard prognostic markers. Despite these advancements, most studies have not incorporated KELIM PSA into multivariable prognostic models in CRPC patients receiving Androgen Receptor Pathway Inhibitor (ARPI) therapy, nor evaluated its prognostic performance relative to clinicopathologic variables such as Gleason score, metastatic sites, or line of ARPI therapy. Researchers have also examined PSA kinetics following androgen receptor signaling inhibitors (ARSIs), finding specific PSA dynamics predictors [9]. Nevertheless, it is still unclear if modeled KELIM PSA parameters offer independent prognostic value when evaluated with current clinical factors. Furthermore, the possibility of using KELIM PSA to improve risk assessment in actual CRPC groups needs more investigation.

Combining traditional prognostic markers with new tools like PSA kinetics is becoming more important as clinical practice advances. Integrating traditional PSA measurements with KELIM PSA and metastatic data could improve risk assessment, helping refine prognostic evaluation and optimize treatment strategies. This research aimed to investigate the prognostic role of KELIM PSA in patients with CRPC treated with ARPI, and to determine whether its predictive value persists alongside established clinical and pathological variables.

## 2. Materials and Methods

### 2.1. Study Design and Patient Population

This retrospective cohort study included 98 patients with castration-resistant prostate cancer (CRPC) who were treated at our institution between May 2016 and May 2025. Eligible patients were required to have histologically confirmed adenocarcinoma of the prostate and progression to CRPC following prior ADT, with or without docetaxel in the castration-sensitive phase. Patients with incomplete clinical or follow-up data were excluded. Electronic medical records at the hospital provided the clinical, demographic, pathological, and treatment data. This study followed the Declaration of Helsinki and was approved by the University Faculty of Medicine Local Ethics Committee (Decision No: 2025/0041, dated 3 July 2025). As this study was retrospective in nature and utilized anonymized data extracted from medical records and laboratory databases, the requirement for informed consent was waived by the institutional ethics committee.

### 2.2. Data Collection and Variables

Baseline demographic characteristics included age, BMI, and smoking status. Collected data included Eastern Cooperative Oncology Group (ECOG) performance status, Gleason/International Society of Urological Pathology (ISUP) score, risk classifications, and history of definitive local treatment before relapse (surgery, radiotherapy, or combined approaches). The castration-sensitive period treatments (ADT or ADT plus docetaxel) and subsequent ARPI therapies (enzalutamide or abiraterone acetate) in CRPC settings were documented. Data on the line of ARPI treatment (first-line vs. second-line in CRPC) were also gathered.

The assessment of disease burden involved documenting metastatic sites: bone, regional lymph nodes, non-regional lymph nodes, lung, and liver. Use of bone-modifying agents was also noted. Laboratory results included lactate dehydrogenase (LDH), evaluated relative to the upper limit of normal (ULN); values ≥ 1.5 × ULN were considered elevated.

### 2.3. PSA Kinetics Modeling and KELIM Calculation

PSA kinetics were modeled according to a log-linear approach derived from the kinetic–pharmacodynamic model described by Carrot et al. [6]. This model assumes that, following treatment initiation, PSA concentrations decline exponentially over time according to a first-order elimination process characterized by the constant KELIM.

For each patient, serum PSA values obtained at baseline and during the first 100 days of therapy were log-transformed to linearize the exponential decline. Patients with missing PSA values within the first 100 days were excluded from the modeling. In cases with occasional missing intermediate values, linear interpolation between adjacent time points was applied. A simple linear regression of log(PSA) versus time was fitted for each patient:

$$C\left(t\right)\;=\;\frac{KPROD}{KELIM}\;\times \;\left(1\;-\;e^{-KELIM\;\times \;t}\right)$$

The individual elimination constant (KELIM) was defined as the negative of the regression slope (KELIM = −β_1_). Patients with at least two valid PSA measurements were included in the analysis.

Individual KELIM estimates were recorded for each patient and subsequently normalized by the population median. For descriptive and survival analyses, KELIM was treated both as a continuous variable and as a categorical variable, classified as favorable (KELIM ≥ 1) or unfavorable (KELIM < 1).

### 2.4. Outcomes

OS was the primary endpoint, defined as the time from initiation of first ARPI therapy in the CRPC setting to death from any cause. Secondary endpoints included PFS, determined as the time from initiation of ARPI therapy to documented disease progression (radiographic or clinical) or death.

### 2.5. Statistical Analysis

All analyses were conducted using R version 4.4.2 (R Foundation for Statistical Computing, Vienna, Austria). The R6 package can be reused to create reusable objective structures, enabling more flexible and modular combinable workflows. The rstatix package was essential for streamlining testing, updating analysis tables, and formatting them for publication.

Inferential statistics are used to draw conclusions about the shares and differences between them. Test selection depended on data normality (assessed with the Shapiro-Wilk test) and test section matching. *T*-tests are used for normally distributed data to compare two independent groups, while ANOVA connects more than two. When the data are not normally distributed, the Wilcoxon rank test is used to connect two groups, or the Kruskal–Wallis test is used to connect more than two groups. Chi-square tests are preferred for categorical data with sufficient cell observations (over five in each cell), but Fisher’s exact tests are used if the model size is small.

Kaplan–Meier curves were used, and the log-rank test compared differences for survival analyses. Cox regression analyses, both univariate and multivariate, were conducted to calculate hazard ratios (HRs) and 95% confidence intervals (CIs) for OS. Variables with a *p*-value < 0.10 in univariate analysis were included. A two-sided *p*-value less than 0.05 was deemed statistically significant.

## 3. Results

### 3.1. Baseline Characteristics

The analysis included 98 patients, with 42 (42.9%) classified as having a favorable KELIM group and 56 (57.1%) as having an unfavorable KELIM PSA group. Table 1 presents a summary of the baseline demographic and clinical features. The mean age of the group was 75.0 ± 8.6 years; no difference existed between groups (*p* = 0.559). Likewise, mean BMI values were comparable (28.3 vs. 27.0 kg/m^2^, *p* = 0.131). There were no significant differences in smoking status, ECOG score distribution, and Gleason/ISUP grading between the groups. Almost half of patients (48.9%) had Gleason 9–10 disease, with comparable distribution among KELIM categories (*p* = 0.473). Likewise, the majority of patients were in ISUP risk categories 4–5 (71.4%), without group differences (*p* = 0.821).

Before relapse, definitive treatment approaches were similar across groups, with surgery ± salvage radiotherapy used in around a quarter of patients (*p* = 0.236). During the castration-sensitive period, most patients were treated with ADT only (65.3%), while 34.7% received ADT and docetaxel. The treatment setting (ADT with/without docetaxel) was not significantly different between KELIM groups (*p* = 0.188).

The use of enzalutamide and abiraterone acetate was similar for CRPC treatment (*p* = 0.465). There was no significant difference in the patterns of ARPI therapy series (first vs. second line) (*p* = 0.397). Most patients had bone metastases (86.7%) and lymph node involvement; visceral metastases were infrequent. Although liver metastases occurred exclusively in the unfavorable KELIM group (5.4%), the difference was not statistically significant (*p* = 0.258). Median OS 29.1 (7.9–87.1) months in the favorable KELIM group vs. 22.6 (4.7–66.6) months in the unfavorable KELIM group (*p* = 0.050). Importantly, a significant difference was found in mortality rates: 62.5% in the unfavorable KELIM group versus 38.1% in the favorable group of patients who died (*p* = 0.029).

### 3.2. Survival Analysis

Univariate Cox regression analyses are shown in Table 2. Many baseline variables, such as BMI, ECOG, ISUP risk category, and metastatic sites, did not significantly relate to OS. Nevertheless, patients who required second-line ARPI treatment in CRPC had a significantly higher mortality risk (HR:2.44, 95% CI:1.39–4.28, *p* = 0.002). Importantly, the KELIM category was also significantly associated with survival: patients in the unfavorable group had more than a twofold increased risk of death compared with those in the favorable group (HR: 2.30, 95%CI: 1.26–4.19, *p* = 0.006).

In the multivariable Cox regression model (Table 3), unfavorable KELIM remained independently associated with shorter OS (HR: 2.09, 95% CI: 1.12–3.89, *p* = 0.020). Additional independent predictors of poor outcome were second-line ARPI therapy (HR: 3.19, 95% CI: 1.71–5.93, *p* < 0.001) and ADT plus docetaxel administered during the CSPC phase (HR: 2.14, 95% CI: 1.11–4.12, *p* = 0.022).

### 3.3. Kaplan–Meier Survival Estimates

**Figure 1:**
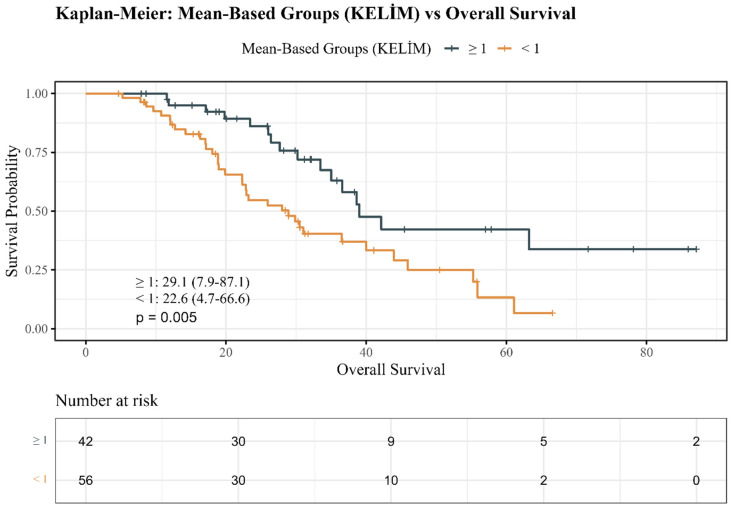
Kaplan–Meier overall survival estimates by KELIM PSA groups.

Kaplan–Meier analyses provided more evidence for the prognostic influence of KELIM PSA category. As shown in Figure 1, OS decreased significantly in unfavorable KELIM patients compared with favorable KELIM (log-rank *p* = 0.005).

**Figure 2:**
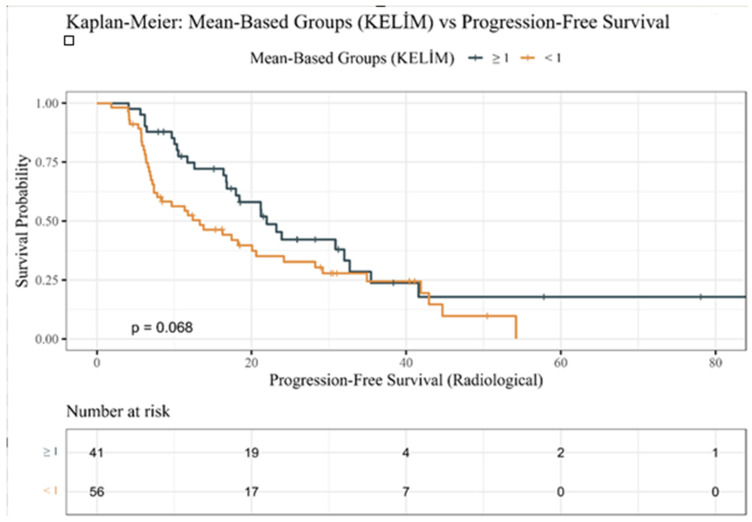
Kaplan–Meier progression-free survival estimates by KELIM PSA groups.

In the same way, PFS curves (Figure 2) demonstrated earlier disease progression in unfavorable KELIM patients but did not reach statistical significance (*p* = 0.068).

## 4. Discussion

The findings of the present study provide valuable evidence on the prognostic significance of KELIM PSA in patients with CRPC treated with ARPIs. Our analysis revealed that unfavorable KELIM PSA (<1) was significantly related to higher mortality and shorter survival compared to favorable KELIM (≥1). Notably, the prognostic effect continued even after adjustment for other variables, confirming the independent role of KELIM PSA in outcome prediction. These results underscore the importance of dynamic PSA monitoring as a key addition to static measures, in line with evidence showing that PSA kinetics are crucial for treatment response and survival.

The prognostic role of KELIM PSA has been explored in prior studies across various therapeutic contexts [6,7,8,9,10,11]. The KELIM model, initially used for ovarian cancer, has been successfully adapted to prostate cancer, assesses the early PSA decline after systemic therapy. KELIM PSA’s ability to predict OS was first demonstrated by Carrot et al. [6] in metastatic prostate cancer patients on ADT ± docetaxel. Carrot et al. [8] provided further validation of prognostic utility in the FIRSTANA trial, showing poorer survival in patients with unfavorable KELIM values, independent of chemotherapy regimen. These results support the current study’s expansion of KELIM PSA’s use to ARPI-treated CRPC groups, validating its use beyond chemotherapy and implying its possible role as a broad prognostic biomarker for systemic treatments. KELIM PSA may serve as a broadly applicable prognostic biomarker across treatment settings, pending confirmation in larger prospective cohorts. Hata et al. [12] also studied PSA decline kinetics and KELIM in docetaxel-treated prostate cancer patients and demonstrated their significant association with survival outcomes. These findings confirm that KELIM’s usefulness in predicting patient outcomes is not limited to hormonal therapies but also extends to chemotherapy, thus supporting its use as a broadly applicable marker of treatment response.

The consistency of these findings across therapeutic settings highlights that KELIM PSA reflects fundamental tumor biology and treatment sensitivity rather than being agent-specific. Rapid PSA declines in patients with favorable KELIM values point to effective PSA-producing tumor clone suppression. Conversely, unfavorable kinetics are linked to aggressive biology, resistant disease, and poor survival outcomes [13]. These observations reinforce the biological results of the current study, supporting KELIM PSA as an early decision-making tool in CRPC management. In line with this, this study highlighted the prognostic impact of PSA for patients with locally advanced and metastatic prostate cancer, supporting the value of ongoing PSA monitoring in multiple clinical contexts [14].

Our findings align with those of Yazgan et al. [11], who evaluated KELIM PSA in 164 metastatic CRPC patients receiving docetaxel as their first-line treatment in the CSPC setting. They reported that OS was significantly lower in patients with unfavorable KELIM (32.0 vs. 46.2 months; HR 1.58, *p* = 0.037). Whereas Yazgan et al. [11] studied patients who were treated with chemotherapy, we found that KELIM PSA maintained its prognostic value in ARPI therapy patients, suggesting that its predictive utility is preserved across different treatment modalities. These findings suggest that KELIM PSA could be used as an indicator of tumor aggressiveness and response to treatment, whether patients are treated with chemotherapy or targeted hormonal agents. In particular, Oka et al. [10] demonstrated PSA kinetics’ applicability to the currently available hormonal treatment options, not only chemotherapy. These findings are consistent with the data of the present study, demonstrating PSA kinetics’ applicability in current hormonal treatments, not only chemotherapy. To our knowledge, the present study is the first to demonstrate the association between KELIM PSA and outcomes in CRPC patients treated with ARPIs.

Survival prediction has also been linked to other PSA kinetic parameters, such as PSA doubling time and time to nadir. Research indicates a connection between poor outcomes and both shorter PSA doubling times and earlier nadirs [13,15]. Furthermore, some studies have also found PSA isoforms to be independent prognostic markers [16]. Fiala et al. showed that baseline total PSA and proenzyme PSA isoforms predicted outcomes in mCRPC patients treated with AR-targeted agents [16]. The findings indicate that PSA kinetics and isoforms could offer additional insights into disease biology and treatment response. However, the present study’s results indicate KELIM PSA could be a more robust and clinically interpretable measure, as it models PSA decline dynamically during the crucial initial treatment phase. This positions KELIM PSA as a complementary tool to existing parameters, potentially offering greater predictive precision.

Our analysis showed that treatment sequencing, in addition to PSA kinetics, significantly impacted prognosis. Patients who required second-line ARPI therapy had a significantly increased mortality risk (HR 16.5). Clinical trial data support the findings of the current study that using ARPI sequentially leads to cross-resistance and less benefit, highlighting the importance of deploying ARPI earlier in the disease course [17]. Hence, the current literature supports the need for optimizing treatment sequence and shows that KELIM PSA is useful in finding patients who might require intensified strategies from the outset.

The presence and distribution of metastases are crucial determinants of prognosis in prostate cancer, reflecting both tumor biology and treatment responsiveness. Bone metastases were most frequent in our study, affecting 86.7% of participants. This observation is consistent with previous findings indicating that osseous involvement represents the dominant metastatic pattern in advanced prostate cancer and is typically associated with a more aggressive disease phenotype and reduced survival [18,19]. Although the unfavorable KELIM group exhibited numerically higher rates of bone and visceral metastases, the difference did not reach statistical significance, possibly due to sample size limitations. Nevertheless, the coexistence of unfavorable KELIM PSA values and widespread skeletal metastases underscores the potential biological link between impaired PSA clearance kinetics and increased tumor burden. Elevated bone turnover and microenvironmental changes induced by metastatic infiltration may further sustain PSA production, thereby contributing to slower PSA elimination rates [6]. These findings highlight that KELIM PSA not only reflects systemic tumor dynamics but also mirrors metastatic behavior, suggesting that integrating both variables could enhance prognostic accuracy. Future studies combining PSA kinetics with detailed radiographic assessments of metastatic sites may clarify how disease distribution modifies the prognostic impact of KELIM PSA in castration-resistant settings.

Among biochemical parameters, ALP and LDH are often considered markers of tumor burden and aggressive disease. Yazgan et al. [11] found that liver metastases and increased LDH independently predicted poor outcomes in docetaxel-treated patients. In our cohort, LDH > 1.5 × ULN was significantly associated with shorter survival in univariate analysis (HR:2.61, 95% CI: 1.33–5.15; *p* = 0.005), but it was not retained in the multivariable model. This finding suggests that elevated LDH may reflect disease aggressiveness, although its independent prognostic impact may be limited when adjusting for dynamic markers such as KELIM [11]. This underlines the importance of nuanced risk that accounts not only for tumor volume but also anatomical and biological variations in metastatic spread. Prognostic assessment has also been proposed using other biomarkers besides PSA kinetics. As an illustration, Hacioglu et al. showed that the Prognostic Nutritional Index provided independent prognostic information in metastatic prostate cancer, highlighting the potential value of integrating systemic and host-related factors into multivariable models [20].

The combined evidence suggests that KELIM PSA offers incremental value over traditional measures such as Gleason score, ISUP risk category, and static PSA levels. Integrating KELIM PSA into clinical practice could enable oncologists might identify poor responders early, tailor treatment intensity, and consider alternative regimens before clinical progression occurs. Furthermore, its applicability across chemotherapy- and ARPI-treated patients supports its potential role as a broadly applicable prognostic tool pending further validation. KELIM PSA represents a promising and widely applicable dynamic biomarker, yet larger multicenter prospective studies are required to confirm its prognostic universality. Prospective studies and prognostic algorithm integration should further explore these clinical implications.

The current study has several strengths. First, it assessed real-world CRPC patients on ARPI, mirroring modern methods and building on studies beyond chemotherapy-treated cohorts. Furthermore, employing multivariable models that consider ECOG status, Gleason/ISUP category, and metastatic sites minimizes the interference and improves the reliability of the results. The Kaplan–Meier survival curves clearly showed outcome differences between KELIM categories. These collective strengths support KELIM PSA in risk stratification to improve care.

Nevertheless, limitations must be acknowledged. Retrospective design inherently introduces bias, and the single-center setting restricts generalizability. The relatively modest sample size, while adequate for exploratory analysis, raises the risk of overfitting and may not fully capture disease heterogeneity. Furthermore, dichotomizing KELIM PSA (<1 vs. ≥1) simplifies interpretation but may obscure prognostic nuances present in continuous modeling. Additionally, biomarkers such as KPROD, Androgen Receptor Splice Variant 7, circulating tumor DNA, or Prostate-Specific Membrane Antigen Positron Emission Tomography imaging, which could enhance predictive accuracy, were not used in the current study [21].

Multicenter, prospective trials are needed to validate the KELIM PSA’s accuracy across different groups and treatment methods. Combining KELIM and KPROD in joint models could improve prediction accuracy [6]. Combining PSA kinetics with genomic and imaging biomarkers might lead to comprehensive, individualized risk models, informing trial stratification, therapeutic sequencing, and adaptive treatment strategies. The development of user-friendly digital tools for real-time KELIM calculation is also needed to enable clinical translation. Based on this perspective, recent reports have emphasized the importance of on-treatment PSA kinetics as a useful biomarker for guiding personalized treatments in metastatic hormone-sensitive prostate cancer, emphasizing its role in follow-up strategies [22]. These efforts have substantial clinical implications; they could transform KELIM PSA from a research parameter to a standard biomarker in prostate cancer precision oncology.

## 5. Conclusions

The current study shows that unfavorable KELIM PSA independently predicts poor survival in CRPC patients on ARPI. The results of the present study align with and expand upon the findings of studies that demonstrate KELIM PSA maintains prognostic value across both chemotherapy- and ARPI-treated populations. KELIM PSA improves prognosis by refining stratification and points to personalized treatment options, including metastatic distribution and treatment sequencing. Prospective studies combining PSA kinetics with molecular and imaging markers are needed to confirm its use and help integrate it into clinical decision-making frameworks.

## Figures and Tables

**Table 1:** Baseline characteristics according to the KELIM PSA level.

	Overall, n (%) n = 98	KELIM PSA ≥ 1, n (%) n = 42	KELIM PSA < 1, n (%) n = 56	*p*
Age (years) *	75.0 ± 8.6	74.4 ± 8.6	75.4 ± 8.7	0.559
BMI (kg/m^2^) *	27.5 ± 4.7	28.3 ± 4.6	27.0 ± 4.7	0.131
BMI				0.242
<30 (kg/m^2^)	68 (69.4)	26 (61.9)	42 (75.0)	
≥30 (kg/m^2^)	30 (30.6)	16 (38.1)	14 (25.0)	
Smoking				0.286
Yes	23 (23.5)	13 (30.9)	10 (17.9)	
No	59 (60.2)	22 (52.4)	37 (66.1)	
Unknown	16 (16.3)	7 (16.7)	9 (16.1)	
ECOG performance score				0.812
0	54 (55.1)	25 (59.5)	29 (51.8)	
1	41 (41.8)	16 (38.1)	25 (44.6)	
2	3 (3.1)	1 (2.4)	2 (3.6)	
Gleason/ISUP Score				0.473
3 + 3	6 (6.1)	4 (9.5)	2 (3.6)	
3 + 4	11 (11.2)	4 (9.5)	7 (12.5)	
4 + 3	11 (11.2)	3 (7.1)	8 (14.3)	
8	22 (22.6)	8 (19.1)	14 (25.0)	
9/10	48 (48.9)	23 (54.8)	25 (44.6)	
Gleason/ISUP Risk Category				0.821
ISUP 1,2,3	28 (28.6)	11 (26.2)	17 (30.4)	
ISUP 4,5	70 (71.4)	31 (73.8)	39 (69.6)	
Previous Definitive Treatment Before Relapse				0.236
No treatment	68 (69.4)	30 (71.4)	38 (67.9)	
Surgery	8 (8.2)	1 (2.4)	7 (12.5)	
RT	6 (6.1)	2 (4.8)	4 (7.1)	
Surgery + Salvage RT	16 (16.3)	9 (21.4)	7 (12.5)	
Treatment in the CSPC period (sensitive)				0.188
ADT	64 (65.3)	31 (73.8)	33 (58.9)	
ADT + Docetaxel	34 (34.7)	11 (26.2)	23 (41.1)	
CRPC ARPI Series				0.397
1st Line	74 (75.5)	34 (81.0)	40 (71.4)	
2nd Line	24 (24.5)	8 (19.0)	16 (28.6)	
CRPC ARPI agent				0.465
Enzalutamide	53 (54.1)	25 (59.5)	28 (50.0)	
Abiraterone acetate	45 (45.9)	17 (40.5)	28 (50.0)	
Metastatic sites				
Bones	85 (86.7)	34 (81.0)	51 (91.1)	0.246
Regional Lymph node	71 (72.5)	30 (71.4)	41 (73.2)	>0.999
Non-regional Lymph node	50 (51.0)	22 (52.4)	28 (50.0)	0.977
Lung	7 (7.1)	2 (4.8)	5 (8.9)	0.695
Liver	3 (3.1)	0 (0.0)	3 (5.4)	0.258
De Novo/Relapse Status				0.678
De Novo	69 (70.4)	31 (73.8)	38 (67.9)	
Relapse	29 (29.6)	11 (26.2)	18 (32.1)	
Use of Bone-Modifying Agents	68 (69.4)	26 (61.9)	42 (75.0)	0.242
Time to Nadir PSA level (months) *	4.78 (−13.5–49.45)	5.22 (0.89–49.45)	4.14 (−13.5–21.36)	0.071
OS (months) *	26.0 (4.7–87.1)	29.1 (7.9–87.1)	22.6 (4.7–66.6)	0.050
Exitus (Yes/No)	51 (52.0)/47 (48.0)	16 (38.1)/26 (61.9)	35 (62.5)/21 (37.5)	0.029

* Numeric variables were presented as median (minimum-maximum) or mean ± standard deviation. Abbreviations: ADT: Androgen Deprivation Therapy, ARPI: Androgen Receptor Pathway Inhibitor, BMI: Body Mass Index, CRPC: Castration-Resistant Prostate Cancer, ECOG: Eastern Cooperative Oncology Group, ISUP: International Society of Urological Pathology, KELIM: Kinetics of ELIMination of PSA (modeled PSA elimination constant), OS: Overall Survival, PSA: Prostate-Specific Antigen, RT: Radiotherapy.

**Table 2:** Univariate analysis of overall survival.

	Univariate		
Characteristic	HR	95% CI	*p*-Value
LDH level			0.005
≤1.5 ULN	—	—	
>1.5 ULN	2.61	1.34–5.08	
CRPC ARPI agent			0.204
Enzalutamide	—	—	
Abiraterone acetate	1.44	0.82–2.53	
BMI			
<30 (kg/m^2^)	—	—	0.439
≥30 (kg/m^2^)	1.27	0.69–2.33	
ECOG			
0	—	—	
1	1.38	0.80–2.40	0.251
2	—	0.00–NA	0.990
Gleason/ISUP Risk Category			0.864
ISUP 1,2,3	—	—	
ISUP 4,5	1.06	0.57–1.96	
De Novo/Relapse Status			
De Novo	—	—	0.200
Relapse	0.67	0.36–1.24	
Treatment During the CSPC Period			
ADT	—	—	0.068
ADT + Docetaxel	1.71	0.96–3.08	
CRPC ARPI Series			0.002
1st Line	—	—	
2nd Line	2.44	1.39–4.28	
Bone metastasis	2.74	0.98–7.61	0.054
Regional Lymph node Metastasis	0.73	0.41–1.28	0.273
Non-Regional Lymph node Metastasis	1.18	0.68–2.05	0.555
Lung Metastasis	1.67	0.59–4.68	0.332
Liver Metastasis	2.64	0.81–8.59	0.106
KELIM category			0.006
Favorable	—	—	
Unfavorable	2.30	1.26–4.19	

Abbreviations: ADT: Androgen Deprivation Therapy, ARPI: Androgen Receptor Pathway Inhibitor, BMI: Body Mass Index, CI: Confidence Interval, CRPC: Castration-Resistant Prostate Cancer, ECOG: Eastern Cooperative Oncology Group, ISUP: International Society of Urological Pathology, KELIM: Kinetics of ELIMination of PSA (modeled PSA elimination constant), LDH: Lactate Dehydrogenase, NA: Not Applicable, ULN: Upper Limit of Normal.

**Table 3:** Multivariate analysis of overall survival.

	Multivariate		
Characteristic	HR	95% CI	*p*-Value
CRPC ARPI Series			
1st Line	—	—	
2nd Line	3.19	1.71–5.93	<0.001
Treatment During the CSPC Period			
ADT	—	—	
ADT + Docetaxel	2.14	1.11–4.12	0.022
KELIM category			
Favorable	—	—	
Unfavorable	2.09	1.12–3.89	0.020

Abbreviations: ADT: Androgen Deprivation Therapy, ARPI: Androgen Receptor Pathway Inhibitor, CI: Confidence Interval, CRPC: Castration-Resistant Prostate Cancer, HR: Hazard Ratio, KELIM: Kinetics of ELIMination of PSA (modeled PSA elimination constant).

## Data Availability

The data that support the findings of this study are available from the corresponding author upon reasonable request.
